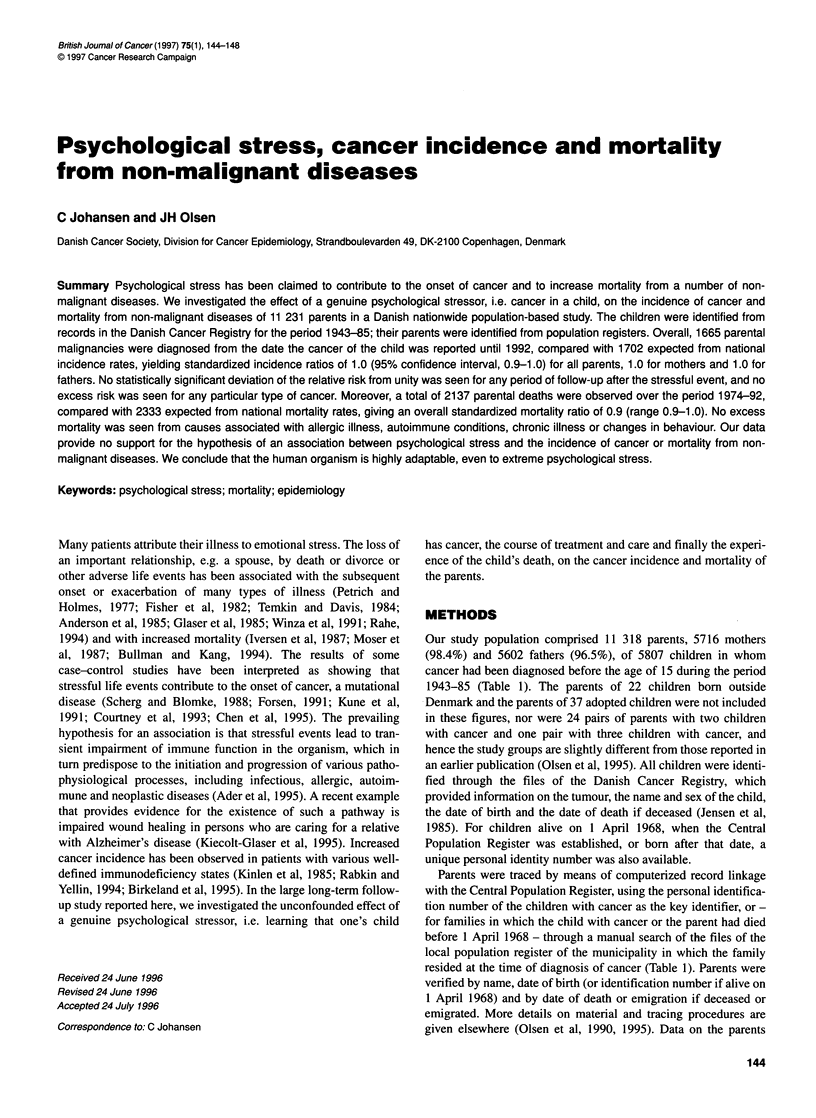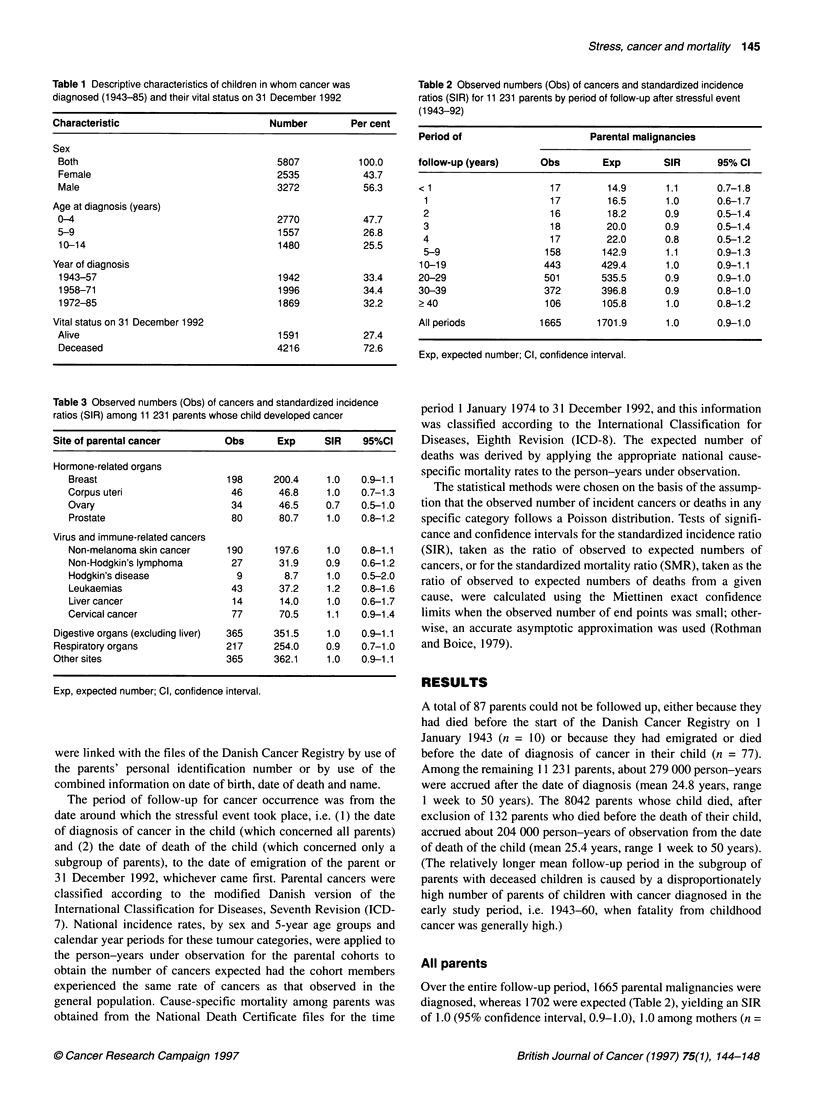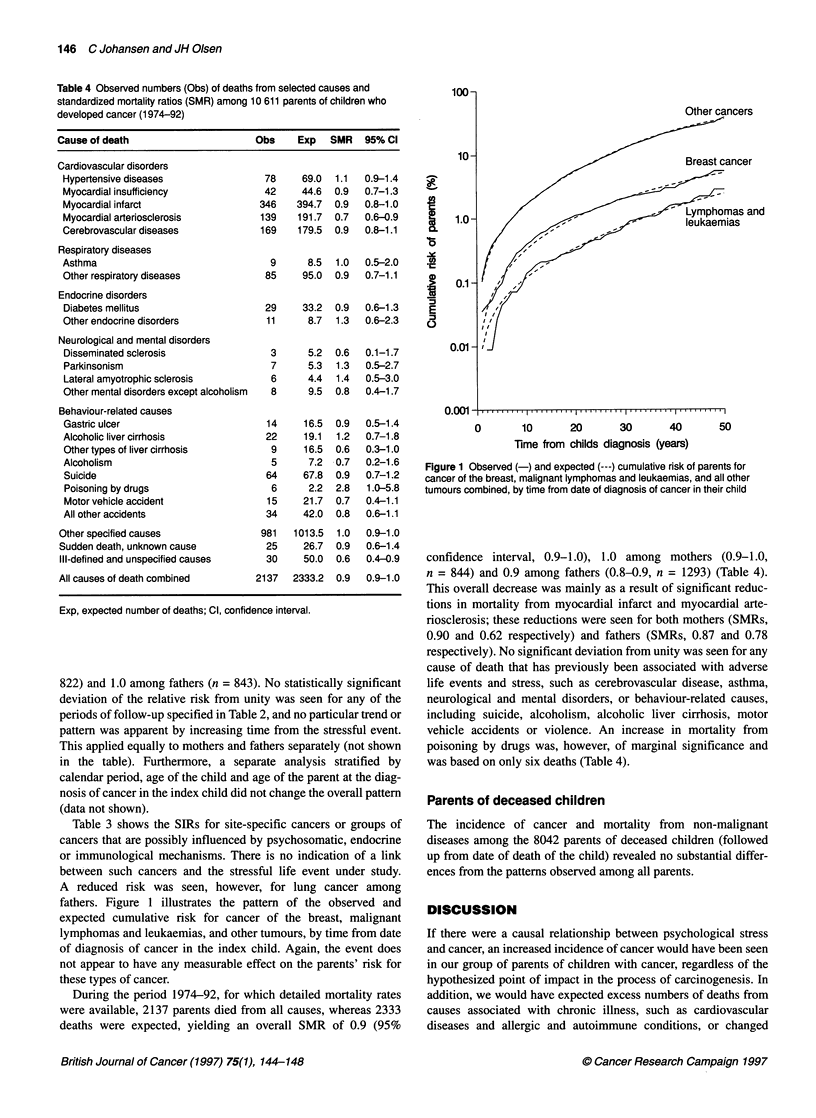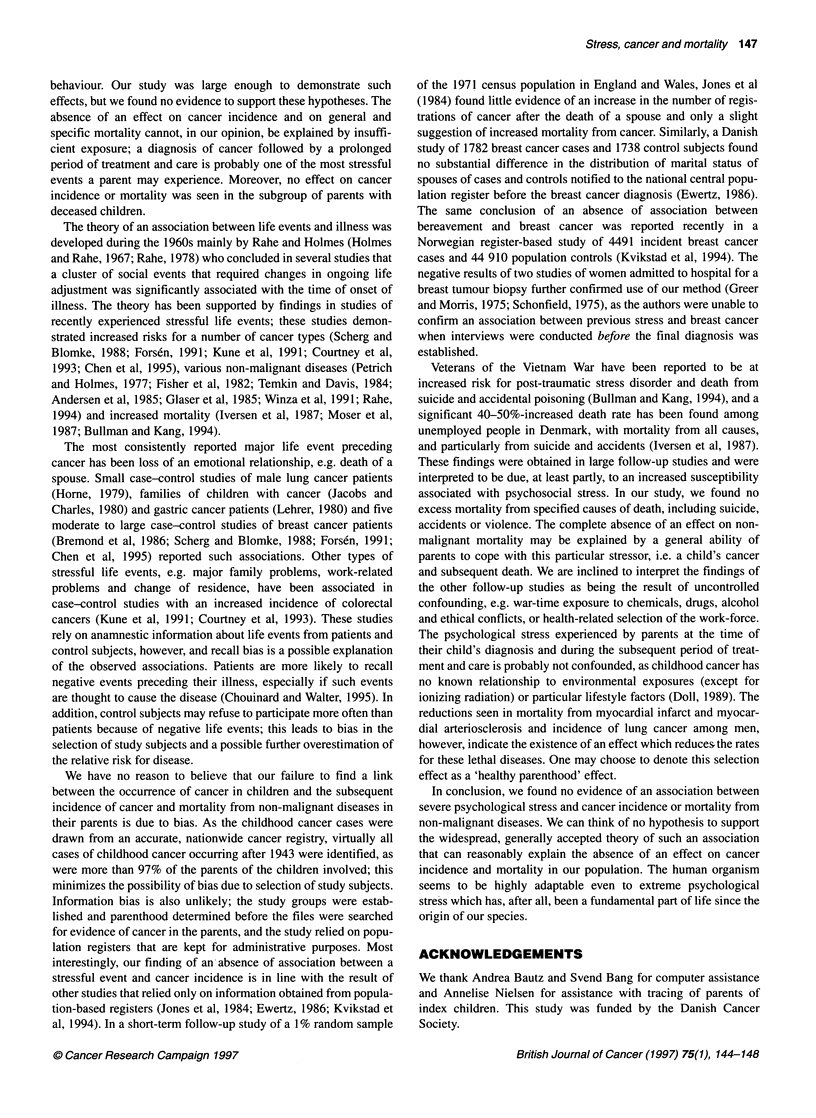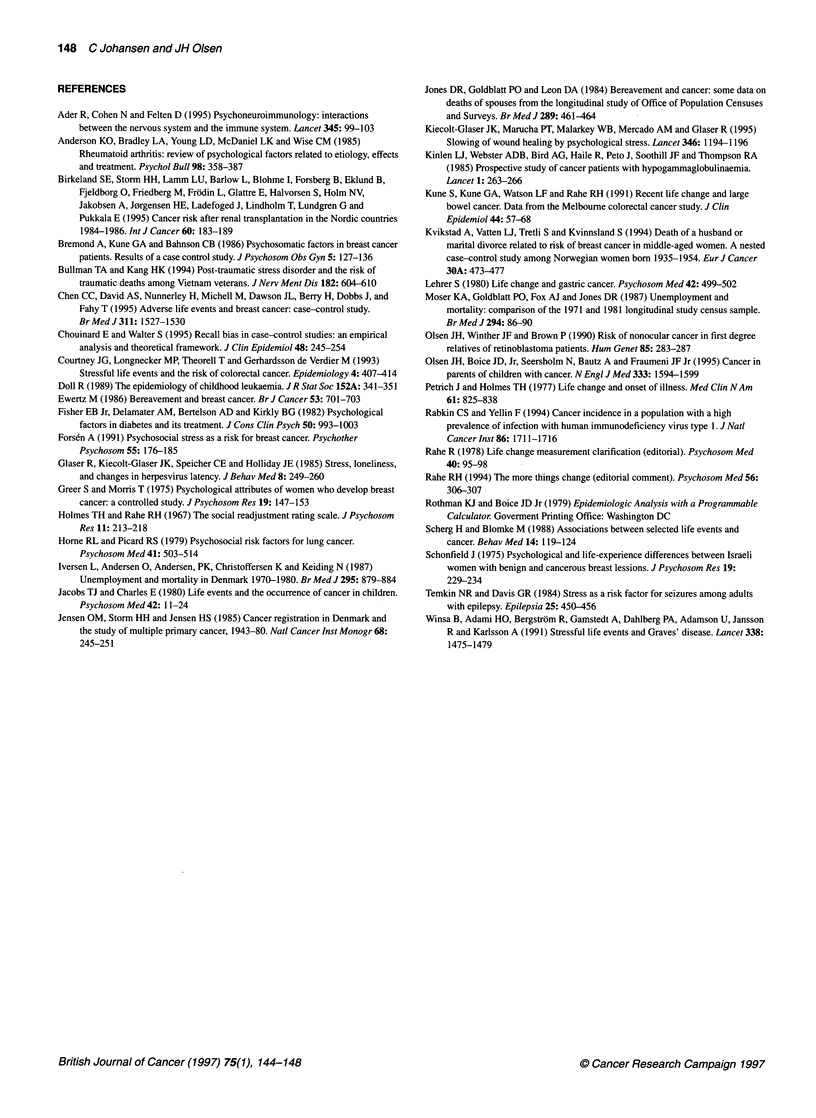# Psychological stress, cancer incidence and mortality from non-malignant diseases.

**DOI:** 10.1038/bjc.1997.24

**Published:** 1997

**Authors:** C. Johansen, J. H. Olsen

**Affiliations:** Danish Cancer Society, Division for Cancer Epidemiology, Copenhagen.

## Abstract

Psychological stress has been claimed to contribute to the onset of cancer and to increase mortality from a number of non-malignant diseases. We investigated the effect of a genuine psychological stressor, i.e. cancer in a child, on the incidence of cancer and mortality from non-malignant diseases of 11,231 parents in a Danish nationwide population-based study. The children were identified from records in the Danish Cancer Registry for the period 1943-85; their parents were identified from population registers. Overall, 1665 parental malignancies were diagnosed from the date the cancer of the child was reported until 1992, compared with 1702 expected from national incidence rates, yielding standardized incidence ratios of 1.0 (95% confidence interval, 0.9-1.0) for all parents, 1.0 for mothers and 1.0 for fathers. No statistically significant deviation of the relative risk from unity was seen for any period of follow-up after the stressful event, and no excess risk was seen for any particular type of cancer. Moreover, a total of 2137 parental deaths were observed over the period 1974-92, compared with 2333 expected from national mortality rates, giving an overall standardized mortality ratio of 0.9 (range 0.9-1.0). No excess mortality was seen from causes associated with allergic illness, autoimmune conditions, chronic illness or changes in behaviour. Our data provide no support for the hypothesis of an association between psychological stress and the incidence of cancer or mortality from non-malignant diseases. We conclude that the human organism is highly adaptable, even to extreme psychological stress.


British Journal of Cancer (1997) 75(1), 144–148
© 1997 Cancer Research Campaign

### Psychological stress, cancer incidence and mortality from non-malignant diseases

**C Johansen and JH Olsen**

Danish Cancer Society, Division for Cancer Epidemiology, Strandboulevarden 49, DK-2100 Copenhagen, Denmark

**Summary** Psychological stress has been claimed to contribute to the onset of cancer and to increase mortality from a number of non-malignant diseases. We investigated the effect of a genuine psychological stressor, i.e. cancer in a child, on the incidence of cancer and mortality from non-malignant diseases of 11 231 parents in a Danish nationwide population-based study. The children were identified from records in the Danish Cancer Registry for the period 1943–85; their parents were identified from population registers. Overall, 1665 parental malignancies were diagnosed from the date the cancer of the child was reported until 1992, compared with 1702 expected from national incidence rates, yielding standardized incidence ratios of 1.0 (95% confidence interval, 0.9–1.0) for all parents, 1.0 for mothers and 1.0 for fathers. No statistically significant deviation of the relative risk from unity was seen for any period of follow-up after the stressful event, and no excess risk was seen for any particular type of cancer. Moreover, a total of 2137 parental deaths were observed over the period 1974–92, compared with 2333 expected from national mortality rates, giving an overall standardized mortality ratio of 0.9 (range 0.9–1.0). No excess mortality was seen from causes associated with allergic illness, autoimmune conditions, chronic illness or changes in behaviour. Our data provide no support for the hypothesis of an association between psychological stress and the incidence of cancer or mortality from non-malignant diseases. We conclude that the human organism is highly adaptable, even to extreme psychological stress.

**Keywords:** psychological stress; mortality; epidemiology

Many patients attribute their illness to emotional stress. The loss of an important relationship, e.g. a spouse, by death or divorce or other adverse life events has been associated with the subsequent onset or exacerbation of many types of illness (Petrich and Holmes, 1977; Fisher et al, 1982; Temkin and Davis, 1984; Anderson et al, 1985; Glaser et al, 1985; Winza et al, 1991; Rahe, 1994) and with increased mortality (Iversen et al, 1987; Moser et al, 1987; Bullman and Kang, 1994). The results of some case–control studies have been interpreted as showing that stressful life events contribute to the onset of cancer, a mutational disease (Scherg and Blomke, 1988; Forsen, 1991; Kune et al, 1991; Courtney et al, 1993; Chen et al, 1995). The prevailing hypothesis for an association is that stressful events lead to transient impairment of immune function in the organism, which in turn predispose to the initiation and progression of various pathophysiological processes, including infectious, allergic, autoimmune and neoplastic diseases (Ader et al, 1995). A recent example that provides evidence for the existence of such a pathway is impaired wound healing in persons who are caring for a relative with Alzheimer's disease (Kiecolt-Glaser et al, 1995). Increased cancer incidence has been observed in patients with various well-defined immunodeficiency states (Kinlen et al, 1985; Rabkin and Yellin, 1994; Birkeland et al, 1995). In the large long-term follow-up study reported here, we investigated the unconfounded effect of a genuine psychological stressor, i.e. learning that one's child has cancer, the course of treatment and care and finally the experience of the child's death, on the cancer incidence and mortality of the parents.

*Received 24 June 1996*
*Revised 24 June 1996*
*Accepted 24 July 1996*

*Correspondence to:* C Johansen

### METHODS

Our study population comprised 11 318 parents, 5716 mothers (98.4%) and 5602 fathers (96.5%), of 5807 children in whom cancer had been diagnosed before the age of 15 during the period 1943–85 (Table 1). The parents of 22 children born outside Denmark and the parents of 37 adopted children were not included in these figures, nor were 24 pairs of parents with two children with cancer and one pair with three children with cancer, and hence the study groups are slightly different from those reported in an earlier publication (Olsen et al, 1995). All children were identified through the files of the Danish Cancer Registry, which provided information on the tumour, the name and sex of the child, the date of birth and the date of death if deceased (Jensen et al, 1985). For children alive on 1 April 1968, when the Central Population Register was established, or born after that date, a unique personal identity number was also available.

Parents were traced by means of computerized record linkage with the Central Population Register, using the personal identification number of the children with cancer as the key identifier, or – for families in which the child with cancer or the parent had died before 1 April 1968 – through a manual search of the files of the local population register of the municipality in which the family resided at the time of diagnosis of cancer (Table 1). Parents were verified by name, date of birth (or identification number if alive on 1 April 1968) and by date of death or emigration if deceased or emigrated. More details on material and tracing procedures are given elsewhere (Olsen et al, 1990, 1995). Data on the parents

**Table 1** Descriptive characteristics of children in whom cancer was diagnosed (1943–85) and their vital status on 31 December 1992

| Characteristic | Number | Per cent |
|---|---|---|
| Sex | | |
| Both | 5807 | 100.0 |
| Female | 2535 | 43.7 |
| Male | 3272 | 56.3 |
| Age at diagnosis (years) | | |
| 0–4 | 2770 | 47.7 |
| 5–9 | 1557 | 26.8 |
| 10–14 | 1480 | 25.5 |
| Year of diagnosis | | |
| 1943–57 | 1942 | 33.4 |
| 1958–71 | 1996 | 34.4 |
| 1972–85 | 1869 | 32.2 |
| Vital status on 31 December 1992 | | |
| Alive | 1591 | 27.4 |
| Deceased | 4216 | 72.6 |

**Table 2** Observed numbers (Obs) of cancers and standardized incidence ratios (SIR) for 11 231 parents by period of follow-up after stressful event (1943–92)

| Period of follow-up (years) | Parental malignancies | | | |
|---|---|---|---|---|
| | Obs | Exp | SIR | 95% CI |
| < 1 | 17 | 14.9 | 1.1 | 0.7–1.8 |
| 1 | 17 | 16.5 | 1.0 | 0.6–1.7 |
| 2 | 16 | 18.2 | 0.9 | 0.5–1.4 |
| 3 | 18 | 20.0 | 0.9 | 0.5–1.4 |
| 4 | 17 | 22.0 | 0.8 | 0.5–1.2 |
| 5–9 | 158 | 142.9 | 1.1 | 0.9–1.3 |
| 10–19 | 443 | 429.4 | 1.0 | 0.9–1.1 |
| 20–29 | 501 | 535.5 | 0.9 | 0.9–1.0 |
| 30–39 | 372 | 396.8 | 0.9 | 0.8–1.0 |
| ≥ 40 | 106 | 105.8 | 1.0 | 0.8–1.2 |
| All periods | 1665 | 1701.9 | 1.0 | 0.9–1.0 |

Exp, expected number; CI, confidence interval.

**Table 3** Observed numbers (Obs) of cancers and standardized incidence ratios (SIR) among 11 231 parents whose child developed cancer

| Site of parental cancer | Obs | Exp | SIR | 95%CI |
|---|---|---|---|---|
| Hormone-related organs | | | | |
| Breast | 198 | 200.4 | 1.0 | 0.9–1.1 |
| Corpus uteri | 46 | 46.8 | 1.0 | 0.7–1.3 |
| Ovary | 34 | 46.5 | 0.7 | 0.5–1.0 |
| Prostate | 80 | 80.7 | 1.0 | 0.8–1.2 |
| Virus and immune-related cancers | | | | |
| Non-melanoma skin cancer | 190 | 197.6 | 1.0 | 0.8–1.1 |
| Non-Hodgkin's lymphoma | 27 | 31.9 | 0.9 | 0.6–1.2 |
| Hodgkin's disease | 9 | 8.7 | 1.0 | 0.5–2.0 |
| Leukaemias | 43 | 37.2 | 1.2 | 0.8–1.6 |
| Liver cancer | 14 | 14.0 | 1.0 | 0.6–1.7 |
| Cervical cancer | 77 | 70.5 | 1.1 | 0.9–1.4 |
| Digestive organs (excluding liver) | 365 | 351.5 | 1.0 | 0.9–1.1 |
| Respiratory organs | 217 | 254.0 | 0.9 | 0.7–1.0 |
| Other sites | 365 | 362.1 | 1.0 | 0.9–1.1 |

Exp, expected number; CI, confidence interval.

were linked with the files of the Danish Cancer Registry by use of the parents' personal identification number or by use of the combined information on date of birth, date of death and name.

The period of follow-up for cancer occurrence was from the date around which the stressful event took place, i.e. (1) the date of diagnosis of cancer in the child (which concerned all parents) and (2) the date of death of the child (which concerned only a subgroup of parents), to the date of emigration of the parent or 31 December 1992, whichever came first. Parental cancers were classified according to the modified Danish version of the International Classification for Diseases, Seventh Revision (ICD-7). National incidence rates, by sex and 5-year age groups and calendar year periods for these tumour categories, were applied to the person–years under observation for the parental cohorts to obtain the number of cancers expected had the cohort members experienced the same rate of cancers as that observed in the general population. Cause-specific mortality among parents was obtained from the National Death Certificate files for the time period 1 January 1974 to 31 December 1992, and this information was classified according to the International Classification for Diseases, Eighth Revision (ICD-8). The expected number of deaths was derived by applying the appropriate national cause-specific mortality rates to the person–years under observation.

The statistical methods were chosen on the basis of the assumption that the observed number of incident cancers or deaths in any specific category follows a Poisson distribution. Tests of significance and confidence intervals for the standardized incidence ratio (SIR), taken as the ratio of observed to expected numbers of cancers, or for the standardized mortality ratio (SMR), taken as the ratio of observed to expected numbers of deaths from a given cause, were calculated using the Miettinen exact confidence limits when the observed number of end points was small; otherwise, an accurate asymptotic approximation was used (Rothman and Boice, 1979).

### RESULTS

A total of 87 parents could not be followed up, either because they had died before the start of the Danish Cancer Registry on 1 January 1943 ($n$ = 10) or because they had emigrated or died before the date of diagnosis of cancer in their child ($n$ = 77). Among the remaining 11 231 parents, about 279 000 person–years were accrued after the date of diagnosis (mean 24.8 years, range 1 week to 50 years). The 8042 parents whose child died, after exclusion of 132 parents who died before the death of their child, accrued about 204 000 person–years of observation from the date of death of the child (mean 25.4 years, range 1 week to 50 years). (The relatively longer mean follow-up period in the subgroup of parents with deceased children is caused by a disproportionately high number of parents of children with cancer diagnosed in the early study period, i.e. 1943–60, when fatality from childhood cancer was generally high.)

### All parents

Over the entire follow-up period, 1665 parental malignancies were diagnosed, whereas 1702 were expected (Table 2), yielding an SIR of 1.0 (95% confidence interval, 0.9–1.0), 1.0 among mothers ($n$ =

© Cancer Research Campaign 1997

**Table 4** Observed numbers (Obs) of deaths from selected causes and standardized mortality ratios (SMR) among 10 611 parents of children who developed cancer (1974–92)

| Cause of death | Obs | Exp | SMR | 95% CI |
|---|---|---|---|---|
| Cardiovascular disorders | | | | |
| Hypertensive diseases | 78 | 69.0 | 1.1 | 0.9–1.4 |
| Myocardial insufficiency | 42 | 44.6 | 0.9 | 0.7–1.3 |
| Myocardial infarct | 346 | 394.7 | 0.9 | 0.8–1.0 |
| Myocardial arteriosclerosis | 139 | 191.7 | 0.7 | 0.6–0.9 |
| Cerebrovascular diseases | 169 | 179.5 | 0.9 | 0.8–1.1 |
| Respiratory diseases | | | | |
| Asthma | 9 | 8.5 | 1.0 | 0.5–2.0 |
| Other respiratory diseases | 85 | 95.0 | 0.9 | 0.7–1.1 |
| Endocrine disorders | | | | |
| Diabetes mellitus | 29 | 33.2 | 0.9 | 0.6–1.3 |
| Other endocrine disorders | 11 | 8.7 | 1.3 | 0.6–2.3 |
| Neurological and mental disorders | | | | |
| Disseminated sclerosis | 3 | 5.2 | 0.6 | 0.1–1.7 |
| Parkinsonism | 7 | 5.3 | 1.3 | 0.5–2.7 |
| Lateral amyotrophic sclerosis | 6 | 4.4 | 1.4 | 0.5–3.0 |
| Other mental disorders except alcoholism | 8 | 9.5 | 0.8 | 0.4–1.7 |
| Behaviour-related causes | | | | |
| Gastric ulcer | 14 | 16.5 | 0.9 | 0.5–1.4 |
| Alcoholic liver cirrhosis | 22 | 19.1 | 1.2 | 0.7–1.8 |
| Other types of liver cirrhosis | 9 | 16.5 | 0.6 | 0.3–1.0 |
| Alcoholism | 5 | 7.2 | 0.7 | 0.2–1.6 |
| Suicide | 64 | 67.8 | 0.9 | 0.7–1.2 |
| Poisoning by drugs | 6 | 2.2 | 2.8 | 1.0–5.8 |
| Motor vehicle accident | 15 | 21.7 | 0.7 | 0.4–1.1 |
| All other accidents | 34 | 42.0 | 0.8 | 0.6–1.1 |
| Other specified causes | 981 | 1013.5 | 1.0 | 0.9–1.0 |
| Sudden death, unknown cause | 25 | 26.7 | 0.9 | 0.6–1.4 |
| Ill-defined and unspecified causes | 30 | 50.0 | 0.6 | 0.4–0.9 |
| All causes of death combined | 2137 | 2333.2 | 0.9 | 0.9–1.0 |

Exp, expected number of deaths; CI, confidence interval.

**Figure 1** Observed (—) and expected (---) cumulative risk of parents for cancer of the breast, malignant lymphomas and leukaemias, and all other tumours combined, by time from date of diagnosis of cancer in their child

822) and 1.0 among fathers ($n$ = 843). No statistically significant deviation of the relative risk from unity was seen for any of the periods of follow-up specified in Table 2, and no particular trend or pattern was apparent by increasing time from the stressful event. This applied equally to mothers and fathers separately (not shown in the table). Furthermore, a separate analysis stratified by calendar period, age of the child and age of the parent at the diagnosis of cancer in the index child did not change the overall pattern (data not shown).

Table 3 shows the SIRs for site-specific cancers or groups of cancers that are possibly influenced by psychosomatic, endocrine or immunological mechanisms. There is no indication of a link between such cancers and the stressful life event under study. A reduced risk was seen, however, for lung cancer among fathers. Figure 1 illustrates the pattern of the observed and expected cumulative risk for cancer of the breast, malignant lymphomas and leukaemias, and other tumours, by time from date of diagnosis of cancer in the index child. Again, the event does not appear to have any measurable effect on the parents' risk for these types of cancer.

During the period 1974–92, for which detailed mortality rates were available, 2137 parents died from all causes, whereas 2333 deaths were expected, yielding an overall SMR of 0.9 (95% confidence interval, 0.9–1.0), 1.0 among mothers (0.9–1.0, $n$ = 844) and 0.9 among fathers (0.8–0.9, $n$ = 1293) (Table 4). This overall decrease was mainly as a result of significant reductions in mortality from myocardial infarct and myocardial arteriosclerosis; these reductions were seen for both mothers (SMRs, 0.90 and 0.62 respectively) and fathers (SMRs, 0.87 and 0.78 respectively). No significant deviation from unity was seen for any cause of death that has previously been associated with adverse life events and stress, such as cerebrovascular disease, asthma, neurological and mental disorders, or behaviour-related causes, including suicide, alcoholism, alcoholic liver cirrhosis, motor vehicle accidents or violence. An increase in mortality from poisoning by drugs was, however, of marginal significance and was based on only six deaths (Table 4).

### Parents of deceased children

The incidence of cancer and mortality from non-malignant diseases among the 8042 parents of deceased children (followed up from date of death of the child) revealed no substantial differences from the patterns observed among all parents.

### DISCUSSION

If there were a causal relationship between psychological stress and cancer, an increased incidence of cancer would have been seen in our group of parents of children with cancer, regardless of the hypothesized point of impact in the process of carcinogenesis. In addition, we would have expected excess numbers of deaths from causes associated with chronic illness, such as cardiovascular diseases and allergic and autoimmune conditions, or changed

© Cancer Research Campaign 1997

behaviour. Our study was large enough to demonstrate such effects, but we found no evidence to support these hypotheses. The absence of an effect on cancer incidence and on general and specific mortality cannot, in our opinion, be explained by insufficient exposure; a diagnosis of cancer followed by a prolonged period of treatment and care is probably one of the most stressful events a parent may experience. Moreover, no effect on cancer incidence or mortality was seen in the subgroup of parents with deceased children.

The theory of an association between life events and illness was developed during the 1960s mainly by Rahe and Holmes (Holmes and Rahe, 1967; Rahe, 1978) who concluded in several studies that a cluster of social events that required changes in ongoing life adjustment was significantly associated with the time of onset of illness. The theory has been supported by findings in studies of recently experienced stressful life events; these studies demonstrated increased risks for a number of cancer types (Scherg and Blomke, 1988; Forsén, 1991; Kune et al, 1991; Courtney et al, 1993; Chen et al, 1995), various non-malignant diseases (Petrich and Holmes, 1977; Fisher et al, 1982; Temkin and Davis, 1984; Andersen et al, 1985; Glaser et al, 1985; Winza et al, 1991; Rahe, 1994) and increased mortality (Iversen et al, 1987; Moser et al, 1987; Bullman and Kang, 1994).

The most consistently reported major life event preceding cancer has been loss of an emotional relationship, e.g. death of a spouse. Small case–control studies of male lung cancer patients (Horne, 1979), families of children with cancer (Jacobs and Charles, 1980) and gastric cancer patients (Lehrer, 1980) and five moderate to large case–control studies of breast cancer patients (Bremond et al, 1986; Scherg and Blomke, 1988; Forsén, 1991; Chen et al, 1995) reported such associations. Other types of stressful life events, e.g. major family problems, work-related problems and change of residence, have been associated in case–control studies with an increased incidence of colorectal cancers (Kune et al, 1991; Courtney et al, 1993). These studies rely on anamnestic information about life events from patients and control subjects, however, and recall bias is a possible explanation of the observed associations. Patients are more likely to recall negative events preceding their illness, especially if such events are thought to cause the disease (Chouinard and Walter, 1995). In addition, control subjects may refuse to participate more often than patients because of negative life events; this leads to bias in the selection of study subjects and a possible further overestimation of the relative risk for disease.

We have no reason to believe that our failure to find a link between the occurrence of cancer in children and the subsequent incidence of cancer and mortality from non-malignant diseases in their parents is due to bias. As the childhood cancer cases were drawn from an accurate, nationwide cancer registry, virtually all cases of childhood cancer occurring after 1943 were identified, as were more than 97% of the parents of the children involved; this minimizes the possibility of bias due to selection of study subjects. Information bias is also unlikely; the study groups were established and parenthood determined before the files were searched for evidence of cancer in the parents, and the study relied on population registers that are kept for administrative purposes. Most interestingly, our finding of an absence of association between a stressful event and cancer incidence is in line with the result of other studies that relied only on information obtained from population-based registers (Jones et al, 1984; Ewertz, 1986; Kvikstad et al, 1994). In a short-term follow-up study of a 1% random sample of the 1971 census population in England and Wales, Jones et al (1984) found little evidence of an increase in the number of registrations of cancer after the death of a spouse and only a slight suggestion of increased mortality from cancer. Similarly, a Danish study of 1782 breast cancer cases and 1738 control subjects found no substantial difference in the distribution of marital status of spouses of cases and controls notified to the national central population register before the breast cancer diagnosis (Ewertz, 1986). The same conclusion of an absence of association between bereavement and breast cancer was reported recently in a Norwegian register-based study of 4491 incident breast cancer cases and 44 910 population controls (Kvikstad et al, 1994). The negative results of two studies of women admitted to hospital for a breast tumour biopsy further confirmed use of our method (Greer and Morris, 1975; Schonfield, 1975), as the authors were unable to confirm an association between previous stress and breast cancer when interviews were conducted *before* the final diagnosis was established.

Veterans of the Vietnam War have been reported to be at increased risk for post-traumatic stress disorder and death from suicide and accidental poisoning (Bullman and Kang, 1994), and a significant 40–50%-increased death rate has been found among unemployed people in Denmark, with mortality from all causes, and particularly from suicide and accidents (Iversen et al, 1987). These findings were obtained in large follow-up studies and were interpreted to be due, at least partly, to an increased susceptibility associated with psychosocial stress. In our study, we found no excess mortality from specified causes of death, including suicide, accidents or violence. The complete absence of an effect on non-malignant mortality may be explained by a general ability of parents to cope with this particular stressor, i.e. a child's cancer and subsequent death. We are inclined to interpret the findings of the other follow-up studies as being the result of uncontrolled confounding, e.g. war-time exposure to chemicals, drugs, alcohol and ethical conflicts, or health-related selection of the work-force. The psychological stress experienced by parents at the time of their child's diagnosis and during the subsequent period of treatment and care is probably not confounded, as childhood cancer has no known relationship to environmental exposures (except for ionizing radiation) or particular lifestyle factors (Doll, 1989). The reductions seen in mortality from myocardial infarct and myocardial arteriosclerosis and incidence of lung cancer among men, however, indicate the existence of an effect which reduces the rates for these lethal diseases. One may choose to denote this selection effect as a 'healthy parenthood' effect.

In conclusion, we found no evidence of an association between severe psychological stress and cancer incidence or mortality from non-malignant diseases. We can think of no hypothesis to support the widespread, generally accepted theory of such an association that can reasonably explain the absence of an effect on cancer incidence and mortality in our population. The human organism seems to be highly adaptable even to extreme psychological stress which has, after all, been a fundamental part of life since the origin of our species.

### ACKNOWLEDGEMENTS

We thank Andrea Bautz and Svend Bang for computer assistance and Annelise Nielsen for assistance with tracing of parents of index children. This study was funded by the Danish Cancer Society.

© Cancer Research Campaign 1997

### REFERENCES

Ader R, Cohen N and Felten D (1995) Psychoneuroimmunology: interactions between the nervous system and the immune system. *Lancet* **345:** 99–103

Anderson KO, Bradley LA, Young LD, McDaniel LK and Wise CM (1985) Rheumatoid arthritis: review of psychological factors related to etiology, effects and treatment. *Psychol Bull* **98:** 358–387

Birkeland SE, Storm HH, Lamm LU, Barlow L, Blohme I, Forsberg B, Eklund B, Fjeldborg O, Friedberg M, Frödin L, Glattre E, Halvorsen S, Holm NV, Jakobsen A, Jørgensen HE, Ladefoged J, Lindholm T, Lundgren G and Pukkala E (1995) Cancer risk after renal transplantation in the Nordic countries 1984–1986. *Int J Cancer* **60:** 183–189

Bremond A, Kune GA and Bahnson CB (1986) Psychosomatic factors in breast cancer patients. Results of a case control study. *J Psychosom Obs Gyn* **5:** 127–136

Bullman TA and Kang HK (1994) Post-traumatic stress disorder and the risk of traumatic deaths among Vietnam veterans. *J Nerv Ment Dis* **182:** 604–610

Chen CC, David AS, Nunnerley H, Michell M, Dawson JL, Berry H, Dobbs J, and Fahy T (1995) Adverse life events and breast cancer: case–control study. *Br Med J* **311:** 1527–1530

Chouinard E and Walter S (1995) Recall bias in case–control studies: an empirical analysis and theoretical framework. *J Clin Epidemiol* **48:** 245–254

Courtney JG, Longnecker MP, Theorell T and Gerhardsson de Verdier M (1993) Stressful life events and the risk of colorectal cancer. *Epidemiology* **4:** 407–414

Doll R (1989) The epidemiology of childhood leukaemia. *J R Stat Soc* **152A:** 341–351

Ewertz M (1986) Bereavement and breast cancer. *Br J Cancer* **53:** 701–703

Fisher EB Jr, Delamater AM, Bertelson AD and Kirkly BG (1982) Psychological factors in diabetes and its treatment. *J Cons Clin Psych* **50:** 993–1003

Forsén A (1991) Psychosocial stress as a risk for breast cancer. *Psychother Psychosom* **55:** 176–185

Glaser R, Kiecolt-Glaser JK, Speicher CE and Holliday JE (1985) Stress, loneliness, and changes in herpesvirus latency. *J Behav Med* **8:** 249–260

Greer S and Morris T (1975) Psychological attributes of women who develop breast cancer: a controlled study. *J Psychosom Res* **19:** 147–153

Holmes TH and Rahe RH (1967) The social readjustment rating scale. *J Psychosom Res* **11:** 213–218

Horne RL and Picard RS (1979) Psychosocial risk factors for lung cancer. *Psychosom Med* **41:** 503–514

Iversen L, Andersen O, Andersen, PK, Christoffersen K and Keiding N (1987) Unemployment and mortality in Denmark 1970–1980. *Br Med J* **295:** 879–884

Jacobs TJ and Charles E (1980) Life events and the occurrence of cancer in children. *Psychosom Med* **42:** 11–24

Jensen OM, Storm HH and Jensen HS (1985) Cancer registration in Denmark and the study of multiple primary cancer, 1943–80. *Natl Cancer Inst Monogr* **68:** 245–251

Jones DR, Goldblatt PO and Leon DA (1984) Bereavement and cancer: some data on deaths of spouses from the longitudinal study of Office of Population Censuses and Surveys. *Br Med J* **289:** 461–464

Kiecolt-Glaser JK, Marucha PT, Malarkey WB, Mercado AM and Glaser R (1995) Slowing of wound healing by psychological stress. *Lancet* **346:** 1194–1196

Kinlen LJ, Webster ADB, Bird AG, Haile R, Peto J, Soothill JF and Thompson RA (1985) Prospective study of cancer patients with hypogammaglobulinaemia. *Lancet* **1:** 263–266

Kune S, Kune GA, Watson LF and Rahe RH (1991) Recent life change and large bowel cancer. Data from the Melbourne colorectal cancer study. *J Clin Epidemiol* **44:** 57–68

Kvikstad A, Vatten LJ, Tretli S and Kvinnsland S (1994) Death of a husband or marital divorce related to risk of breast cancer in middle-aged women. A nested case–control study among Norwegian women born 1935–1954. *Eur J Cancer* **30A:** 473–477

Lehrer S (1980) Life change and gastric cancer. *Psychosom Med* **42:** 499–502

Moser KA, Goldblatt PO, Fox AJ and Jones DR (1987) Unemployment and mortality: comparison of the 1971 and 1981 longitudinal study census sample. *Br Med J* **294:** 86–90

Olsen JH, Winther JF and Brown P (1990) Risk of nonocular cancer in first degree relatives of retinoblastoma patients. *Hum Genet* **85:** 283–287

Olsen JH, Boice JD, Jr, Seersholm N, Bautz A and Fraumeni JF Jr (1995) Cancer in parents of children with cancer. *N Engl J Med* **333:** 1594–1599

Petrich J and Holmes TH (1977) Life change and onset of illness. *Med Clin N Am* **61:** 825–838

Rabkin CS and Yellin F (1994) Cancer incidence in a population with a high prevalence of infection with human immunodeficiency virus type 1. *J Natl Cancer Inst* **86:** 1711–1716

Rahe R (1978) Life change measurement clarification (editorial). *Psychosom Med* **40:** 95–98

Rahe RH (1994) The more things change (editorial comment). *Psychosom Med* **56:** 306–307

Rothman KJ and Boice JD Jr (1979) *Epidemiologic Analysis with a Programmable Calculator.* Goverment Printing Office: Washington DC

Scherg H and Blomke M (1988) Associations between selected life events and cancer. *Behav Med* **14:** 119–124

Schonfield J (1975) Psychological and life-experience differences between Israeli women with benign and cancerous breast lessions. *J Psychosom Res* **19:** 229–234

Temkin NR and Davis GR (1984) Stress as a risk factor for seizures among adults with epilepsy. *Epilepsia* **25:** 450–456

Winsa B, Adami HO, Bergström R, Gamstedt A, Dahlberg PA, Adamson U, Jansson R and Karlsson A (1991) Stressful life events and Graves' disease. *Lancet* **338:** 1475–1479

© Cancer Research Campaign 1997